# Efficient Extraction of Carotenoids from *Sargassum muticum* Using Aqueous Solutions of Tween 20

**DOI:** 10.3390/md17050310

**Published:** 2019-05-25

**Authors:** Flávia A. Vieira, Sónia P. M. Ventura

**Affiliations:** 1 EMarT Group—Emerging Materials Research and Technologies—School of Design, Management and Production Technologies Northern Aveiro-ESAN, University of Aveiro, Estrada do Cercal, 449, Oliveira de Azeméis, 3720-509 Aveiro, Portugal; flavia.vieira@ua.pt; 2 Department of Chemistry, Aveiro Institute of Materials—CICECO, University of Aveiro, Campus Universitário de Santiago, 3810-193 Aveiro, Portugal

**Keywords:** macroalgae, *Sargassum muticum*, carotenoids, non-ionic surfactant, aqueous solutions, Tween 20

## Abstract

The replacement of synthetic compounds by natural products witnesses an increasing demand from the pharmaceutical, cosmetic, food and nutraceutical industries. Included in the set of natural raw materials that are poorly explored are the macroalgae. Despite the detailed characterization and identification of most relevant biomolecules that are present in the main macroalgae species, there remains a lack of efficient and economically viable processes available to meet the needs of the markets. In this work, an efficient and single-step process, based on aqueous solutions of Tween 20, to recover carotenoids from *Sargassum muticum*, an invasive brown macroalgae species present in the Portuguese coast, is proposed and optimized allowing an extraction yield of 2.78 ± 0.4 mg_carotenoids_.g_dried mass_^−1^, which is shown to increase the extraction efficiency by 38% when compared with traditional methods.

## 1. Introduction

Approximately 25 million tonnes of seaweeds are harvested annually. These are in general processed as cosmetics, fertilizers, and food [1]. In the last few years, macroalgae become one of the most promising natural resources, not only because they are an alternative biomass for sustainable energy production, but also for solar energy conversion [2] and biofuel production [3,4]. Currently, and due to the concerns with the economic viability of the energy-oriented processes, other approaches are increasing their importance on the field of marine resources. In this sense, many works are being developed focusing both on microalgae [5] and macroalgae [6] as natural sources of added-value compounds. Among these are the carotenoids and, in particular, the fucoxanthin [7]. Fucoxanthin is a very sensitive molecule [8], easily affected by temperature and light [9], recognized by its interesting physiological functions and biological properties, which are essential for human health [10,11,12,13,14,15,16,17]. There are different natural sources of this pigment; however, brown seaweeds and particularly *Sargassum muticum* (*S. muticum*) represent the biomass where this specific carotenoid is more abundant. *S. muticum* has its origin in Japan, and thus it is considered an invasive species in Europe. In the coast of Portugal [18], it is common to find *S. muticum* north of the Mondego river. The north of Portugal is a biogeographic transition zone where many species of macroalgae have their distribution limits, which makes this region particularly interesting in terms of the commercial exploration of algal bioproducts and materials. 

Aqueous solutions of surfactants have been recognized, by us and others [19,20], as promising solvents to be used in the recovery of hydrophobic compounds from a large range of raw materials. In some previous works, we investigated the use of non-ionic and ionic surfactants in aqueous media, and good results were obtained with regards to the extraction of carotenoids. However, the approaches previously reported showed poor results in terms of selectivity [21,22]. In this context, this work investigated the use of aqueous solutions of polysorbate 20 (polyoxyethylene sorbitan monolaurate), currently designated as Tween 20. This is a common surfactant extensively used in the chemical industry; it is biocompatible and recognized as a good stabilizing agent for proteins [23,24,25]. Here, aqueous solutions of Tween 20 were tested for the extraction of fucoxanthin, the most abundant carotenoid present in the biomass; furthermore, the concentration of the surfactant (C_surf_), solid-liquid ratio (R_(S/L)_) and time of extraction (t) were optimized.

## 2. Results and Discussion

**Figure 1:**
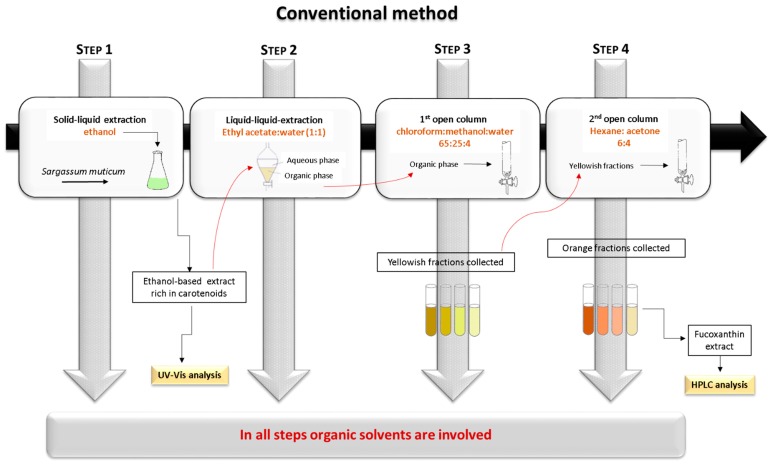
Scheme of the conventional method used for the extraction of the carotenoids and the purification of the fucoxanthin.

To accomplish the main objective of this work, a novel methodology for the (solid-liquid) extraction of carotenoids from *S. muticum* is here proposed and optimized. In this work, the conventional methodology [26], depicted in Figure 1, was applied. This methodology comprises several steps, where various organic solvents and/or mixtures of organic solvents are used to extract the carotenoids, and, in particular, the fucoxanthin. In addition to the complexity of this process of extracting the carotenoids, an extra step for drying the biomass is normally added. Despite the fact that, in this work, dry biomass samples were used, this step can be skipped with the alternative methodology here envisioned, and the biomass can be used as it was collected from nature. When the conventional methodology was applied, the yield of extraction of the carotenoids was 1.87 ± 0.02 mg_carotenoids_.g_dried mass_^−1^, and the content of the fucoxanthin was 0.100 ± 0.004 mg_fucoxanthin_.g_dried mass_^−1^. The yield of extraction obtained with the traditional method was further used as a benchmark to evaluate the performance of the alternative process developed in this work.

**Figure 2:**
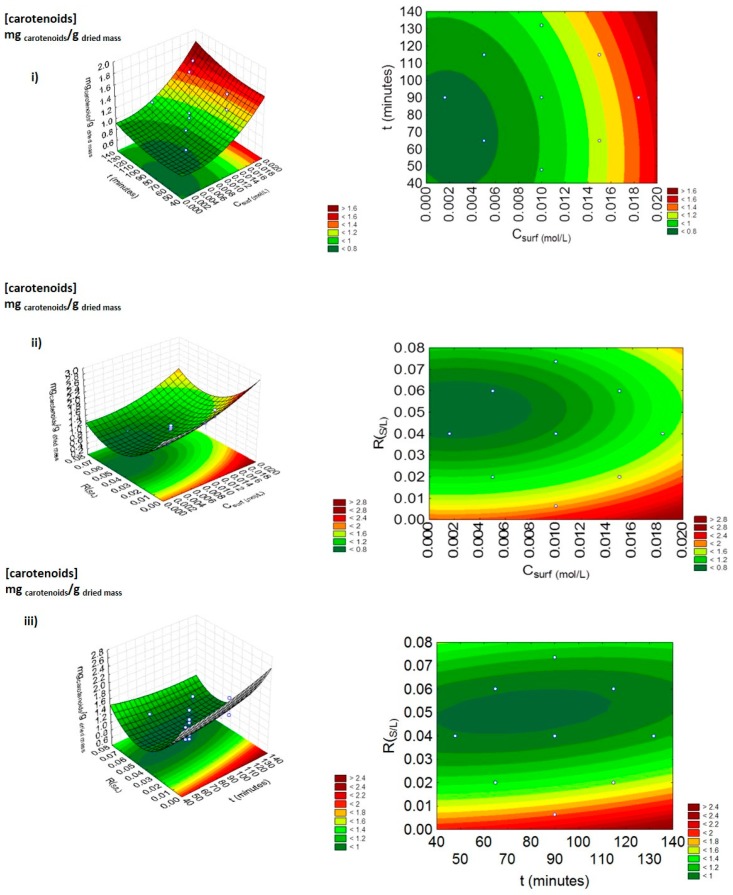
Surface response plots (left) and contour plots (right) on the yield of extraction of the carotenoids (mg_carotenoids_.g_dried mass_^−1^) by combining the effects of (**i**) C_surf_ (mol.L^−1^) and t (minutes), (**ii**) C_surf_ (mol.L^−1^) and R_(S/L)_, and (**iii**) R_(S/L)_ and t (minutes), using aqueous solutions of Tween 20.

Here, aqueous solutions of Tween 20 were investigated. The optimization started by testing this solvent in the same conditions adopted from our previous works [21], namely: concentration of surfactant (C_surf_) = 0.01 mol.L^−1^, solid-liquid ratio - R_(S/L)_ = 0.04 and time of extraction (t) = 90 min. On this first assay, the yield of extraction obtained was 1.38 ± 0.02 mg_carotenoids_.g_dried mass_^−1^, a good result obtained when compared with the performance of the aqueous solutions of other non-ionic and ionic surfactants previously studied in the same conditions [21,22]. Then, the effect of the process conditions fixed in the first assay (C_surf_, R_(S/L)_ and t) was investigated through the application of a factorial planning 2^3^. The un-coded and coded coefficients representing the central, factorial, and axial points used are presented in Table S1 (Supplementary Materials). The yield of extraction of the carotenoids experimentally obtained and the predicted values, as well as the statistical analyses performed, are presented in the Supporting Information (Tables S2–S4 of the Supplementary Material). The response surface and the contour plots for Tween 20 are represented in Figure 2. The data obtained and the statistical analysis carried out suggest that the central point was not correctly selected, since the area of maximum yield of extraction of the carotenoids is not included in the intervals considered mainly in terms of C_surf_ and R_(S/L)_. No significant differences (*p*-value 0.05) were identified between the predicted and experimental values that were obtained (Figure S1 and Tables S2–S4 in the Supplementary materials), showing the accuracy and precision of the model equations obtained for this factorial planning (data presented in Table S3). 

**Figure 3:**
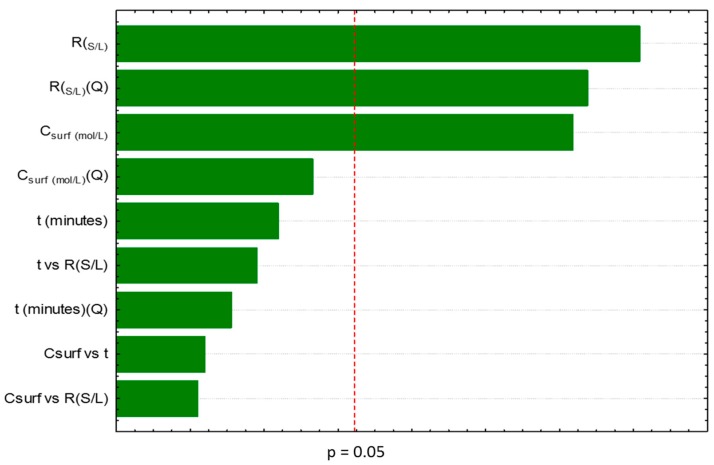
Pareto chart obtained for the factorial planning 2^3^ obtained for the study of aqueous solutions of Tween 20.

Moreover, from the results depicted in Figure 2, it was possible to conclude that *t* is not an important parameter for the carotenoids extraction (considered as non-statistically relevant), contrarily to what happens with the R_(S/L)_, its quadratic function and the C_surf_, a result also supported by the Pareto chart depicted in Figure 3.

**Figure 4:**
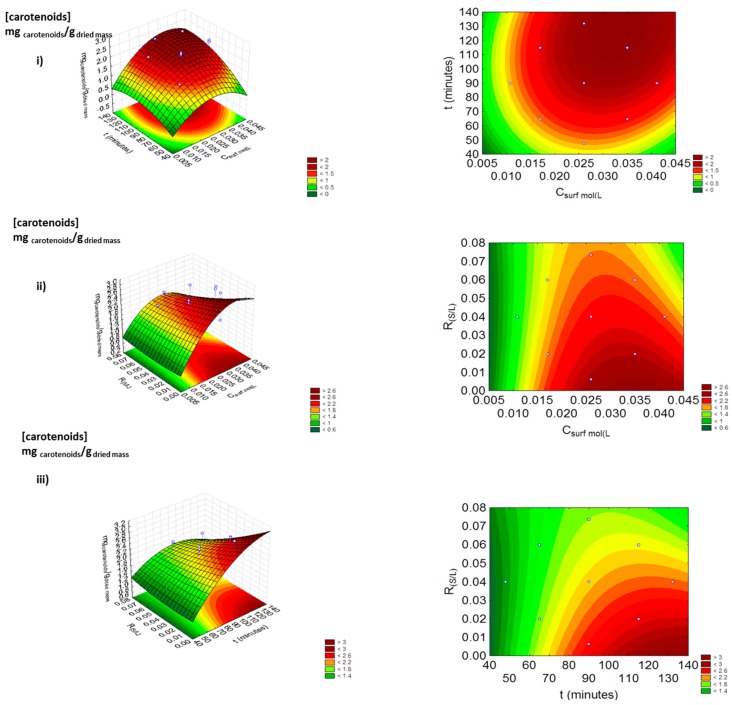
Second factorial planning 2^3^: surface response plots (left) and contour plots (right) on the yield of extraction of the carotenoids (mg_carotenoids_.g_dried mass_^−1^) by combining the effects of (**i**) C_surf_ (mol.L^−1^) and t (minutes), (**ii**) C_surf_ (mol.L^−1^) and R_(S/L)_, and (**iii**) R_(S/L)_ and t (minutes), using aqueous solutions of Tween 20.

Since this study led to an undefined optimum for the yield of extraction of the carotenoids, a new factorial planning was carried out, taking into account the best results found in the first factorial planning as the central point, C_surf_ = 0.026 mol.L^−1^, t = 90 min (since it was previously defined as a non-significant parameter) and R_(S/L)_ = 0.04. The experimental design and statistical analysis were developed, and the accuracy and precision of the model equations were determined, being the main results shown in Tables S5–S7 and Figure S2 in the Supplementary Material. Figure 4 depicts the effect of the three variables studied in the concentration of the carotenoids extracted in a single-step extraction. 

**Figure 5:**
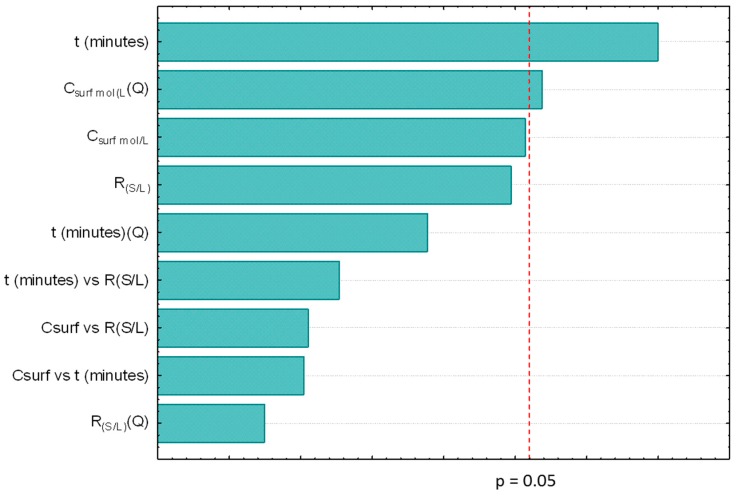
Pareto chart obtained for the second factorial planning 2^3^ using aqueous solutions of Tween 20.

The results obtained in the second factorial planning and depicted in Figure 4 show the impact of the variables under study for the new intervals that were selected. By analyzing the response surface and contour plots, we concluded that the most significant variables studied were the time of extraction, followed by the concentration of Tween 20, which is also corroborated by the results depicted in the Pareto chart (Figure 5). When the time of extraction increases, the contact between the cells of the macroalgae and the solvent is improved, allowing the disruption of the cells in a more efficient way [27], but it can also promote the increase in the solubility of the carotenoids in the aqueous solution [22,27,28]. By increasing the concentration of the surfactant, more micelles are formed, helping to increase the solubility potential of the aqueous solution for the carotenoids [22,29].

Through the analysis of the second factorial planning, and despite the slightly higher discrepancy observed between the experimental and predicted results attributed to the heterogeneity of the raw material, it was possible to define the optimum conditions as C_surf_ = 0.046 mol.L^−1^, t = 140 min and R_(S/L)_ = 0.02. This process improved the extraction of the carotenoids by more 38% when compared with the conventional methodology using ethanol (yield of extraction = 1.87 ± 0.02 mg_carotenoids_.g_dried mass_^−1^). By applying the optimized conditions, it was possible to achieve a maximum extraction of the carotenoids of 2.78 ± 0.4 mg_carotenoids_.g_dried mass_^−1^, the best result obtained so far in the use of aqueous solutions of non-ionic surfactants [21]. 

## 3. Materials and Methods 

### 3.1. Materials

The macroalgae that were used were collected from the Portuguese coast by the company ALGAplus (Ílhavo, Portugal). The biomass was oven-dried with a continuous ventilation and controlled temperature of 40 °C until a constant weight was obtained. The dry biomass samples were stored in the dark, then transported to the University of Aveiro facilities and stored at −20 °C for further use.

In this work, ethanol (Fisher Scientific, Loughborough, UK), methanol (purity 100%, CHEM-LAB, Zedelgem, Belgium), ethyl acetate (purity 99%, VWR BDH-Prolabor, Alfragide, Portugal), chloroform (purity 99%, Carlo Erba, Chaussée du Vexin, France), n-hexane (HPLC grade, Carlo Erba, Chaussée du Vexin, France), acetone (purity 100%, VWR Normapur, Alfragide, Portugal) and acetonitrile of HPLC grade (Fisher Chemical, Loughborough, UK) were applied. The column of chromatography included in the conventional approach was prepared with silica gel G-60 (Sigma-Aldrich, St. Louis, MO, USA). A commercial standard of fucoxanthin (purity ≥ 95%) and Tween 20 (purity ≥ 97%) were acquired from (Sigma-Aldrich, St. Louis, MO, USA). 

### 3.2. Conventional Extraction 

A conventional ethanolic extraction approach adapted from the literature was applied in this work for comparative purposes, following exactly the same experimental methodology [26]. 

### 3.3. Optimization of the Alternative Method Using Aqueous Solutions of Tween 20

The experimental methodology applied to test the effect of the aqueous solutions of Tween 20, as well as the influence of the different process conditions, followed the optimization methodology described in our previous works [21,22]. The yield of the carotenoids extraction was assessed in triplicate, being the results presented as the average of the three experiments (mg_carotenoids_.g_dried mass_^−1^). The optimization of the concentration of Tween 20 (C_surf_), time of extraction (t) and solid-liquid ratio [R_(S/L)_] was carried out by applying a 2^3^ factorial planning, as previously described [21,22]. The adequacy of the model was determined [30]. Three-dimensional surface response plots were originated by varying two variables within the experimental range and fixing all other factors at the central point. Each factorial planning that was developed used a central point, which was experimentally assessed at least in triplicate. An additional 12 to 20 experiments for each factorial planning were performed, representing the various processing conditions that were repeated to guarantee the accuracy of the data when necessary. The Statsoft Statistica 8.0© software Statsoft© (Statsoft, Round Rock, TX, USA) was applied in the statistical analysis, representing the response surfaces and contour plots developed with the same software.

### 3.4. Carotenoids Quantification 

After obtaining the ethanol-based solutions that were rich in carotenoids, aliquots of 20 µL of each fraction were analyzed by a UV–Vis spectrophotometer (SHIMADZU, UV-1700 Pharma Spec, Kyoto, China). Considering the previous characterization of the whole spectrum from 350 to 700 nm, the carotenoids content was measured at 417 nm. Moreover, the fucoxanthin content in the different samples was measured by HPLC (Shimadzu LC-10A, Kyoto, China) equipped with a reversed phase C18 column (Vydac 201TP54, 250 mm × 4.6 mm internal diameter), coupled to a precolumn (Vydac 218GK54, 5 µM) with a detector operating at 470 nm. The carotenoids were quantified for the total amount of carotenoids (in mg_carotenoids_/g_dried mass_).

## 4. Conclusions

New processes of extraction are required for the recovery of high-added value compounds from natural sources. The marine biomass is a very good example of a raw material with a high industrial potential, but that is being underexplored. In this work, the development of a single process of extraction of carotenoids from a brown invasive macroalgae using aqueous solutions of Tween 20 was studied. Through the proper optimization of the process conditions, namely the solid-liquid ratio (R_(S/L)_ = 0.02), concentration of surfactant (C_surf_ = 0.046 mol.L^−1^) and time of extraction (t = 140 min), our previous results, which used aqueous solutions of non-ionic surfactants to extract carotenoids from the dried biomass, were largely surpassed. Through this work, a simple and more sustainable process, able to extract 2.78 ± 0.4 mg_carotenoids_.g_dried mass_^−1^, was designed; this was 38% more efficient than the traditional method that used ethanol (yield of extraction = 1.87 ± 0.02 mg_carotenoids_.g_dried mass_^−1^).

## Supplementary Materials

The following are available online at https://www.mdpi.com/1660-3397/17/5/310/s1, Figure S1: Graph for the predicted values *versus* observed values for Tween 20, 1st RSM, Figure S2: Graph for the predicted values *versus* observed values for Tween 20, 2nd RSM, Table S1: 2^3^ factorial planning for Tween 20, Table S2: Data attributed to the independent variables [C_surf_, t and R_(S/L)_] to define the 2^3^ factorial planning for 1st RSM for Tween 20 and the respective results of the concentration of carotenoids extracted experimentally, the theoretical results found for the mathematical model developed and the respective relative deviation, Table S3: Regression coefficients of the predicted second-order polynomial model for the carotenoids extraction obtained from the 1st RSM design using the Tween 20 aqueous solution, Table S4: ANOVA data for the extraction of the carotenoids obtained from the factorial planning carried out with Tween 20, Table S5: Data attributed to the independent variables [Cs_urf_, t and R_(S/L)_] to define the 2^3^ factorial planning for 2nd RSM for Tween 20 and the respective results of the concentration of carotenoids extracted experimentally, the theoretical results found for the mathematical model developed and the respective relative deviation, Table S6: Regression coefficients for the 2nd 2^3^ factorial planning with the surfactant Tween 20 aqueous solution, Table S7: ANOVA data for the extraction of the carotenoids obtained from the 2nd factorial planning carried out with Tween 20.
