# Supplementary material for: Efficient Extraction of Carotenoids from Sargassum muticum Using Aqueous Solutions of Tween 20
Source: Mar Drugs. 2019 May 25;17(5):310. doi: 10.3390/md17050310 (PMC6562716; doi:10.3390/md17050310)
Supplement: Supplementary file 1 [file marinedrugs-17-00310-s001.pdf]

Supporting Materials

Efficient Extraction of Carotenoids from *Sargassum muticum* Using Aqueous Solutions of Tween 20

Flávia A. Vieira [1] and Sónia P. M. Ventura [2,*]

[1] EMarT Group –Emerging Materials Research and Technologies - School of Design, Management and Production Technologies Northern Aveiro- ESAN, University of Aveiro, Estrada do Cercal, 449, 3720-509 Oliveira de Azeméis, Aveiro, Portugal; flavia.vieira@ua.pt

[2] Department of Chemistry, Aveiro Institute of Materials - CICECO, University of Aveiro, Campus Universitário de Santiago, 3810-193 Aveiro, Portugal

* Correspondence: spventura@ua.pt; Tel: +351-234-370200; Fax: +351-234-370084

Table S1. 2^3 factorial planning for Tween 20.

Experiment	X1	X2	X3
1	-1	-1	-1
2	1	-1	-1
3	-1	1	-1
4	1	1	-1
5	-1	-1	1
6	1	-1	1
7	-1	1	1
8	1	1	1
9	-1.68	0	0
10	1.68	0	0
11	0	-1.68	0
12	0	1.68	0
13	0	0	-1.68
14	0	0	1.68
15	0	0	0
16	0	0	0
17	0	0	0
18	0	0	0
19	0	0	0
20	0	0	0

Table S2. Data attributed to the independent variables [C_{surf}, t and $R_{(S/L)}$] to define the 2^3 factorial planning for 1st RSM for Tween 20 and respective results of concentration of carotenoids extracted experimentally, the theoretical results found for the mathematical model developed and the respective relative deviation.

Experiment	C_{surf} (mol.L^{-1})	t (minutes)	$R_{(S/L)}$	Yield of extraction ($mg_{carotenoids}.g_{dried\ mass}^{-1}$)$_{experimental}$	Yield of extraction ($mg_{carotenoids}.g_{dried\ mass}^{-1}$)$_{theoretical}$	Residues
1	0.005	65	0.02	1.23	1.16	0.07
2	0.015	65	0.02	1.54	1.55	-0.01
3	0.005	115	0.02	1.33	1.32	0.00
4	0.015	115	0.02	1.76	1.75	0.01
5	0.005	65	0.06	0.88	0.80	0.08
6	0.015	65	0.06	1.26	1.16	0.09
7	0.005	115	0.06	0.88	0.78	0.11
8	0.015	115	0.06	1.22	1.18	0.03
9	0.0016	90	0.04	0.66	0.77	-0.11
10	0.0184	90	0.04	1.41	1.44	-0.03
11	0.01	48	0.04	0.82	0.91	-0.09
12	0.01	132	0.04	1.02	1.07	-0.04
13	0.01	90	0.01	1.89	1.89	0.00
14	0.01	90	0.07	0.97	1.11	-0.14
15	0.01	90	0.04	1.08	0.93	0.15
16	0.01	90	0.04	0.83	0.93	-0.11
17	0.01	90	0.04	0.77	0.93	-0.16
18	0.01	90	0.04	0.83	0.93	-0.10
19	0.01	90	0.04	1.17	0.93	0.23
20	0.01	90	0.04	0.94	0.93	0.01

Table S3. Regression coefficients of the predicted second-order polynomial model for the carotenoids extraction obtained from the 1st RSM design using Tween 20 aqueous solution.

	Regression Coefficients	Standard deviation	t-student (10)	p - value
interception	1.88	0.77	2.44	0.03
C_{surf}	-148.48	513.89	-0.29	0.78
t	0.00	0.01	-0.11	0.91
$R_{(S/L)}$	-43.13	12.85	-3.36	0.01
C_{surf}^2	246925.59	149114.52	1.66	0.13
t^2	0.00	0.00	0.56	0.59
$R_{(S/L)}^2$	501.20	93.20	5.38	0.00
C_{surf} * t	0.79	4.00	0.20	0.85
C_{surf} * $R_{(S/L)}$	-512.82	4995.45	-0.10	0.92
t * $R_{(S/L)}$	-0.09	0.10	-0.90	0.39

Table S4. ANOVA data for the extraction of carotenoids obtained from the factorial planning carried with Tween 20.

	Sum Squares	Degrees of Freedom	Mean Square	Fcal	p -value
Regression	1.92	9	0.21	10.70	0.00
Error	0.20	10	0.02		
Total	2.12				

Table S5. Data attributed to the independent variables [C_{surf}, t and $R_{(S/L)}$] to define the 2^3 factorial planning for 2nd RSM for Tween 20 and respective results of concentration of carotenoids extracted experimentally, the theoretical results found for the mathematical model developed and the respective relative deviation.

Experiment	C_{surf} (mol.L^{-1})	t (minutes)	$R_{(S/L)}$	Yield of extraction ($mg_{carotenoids}.g_{dried\ mass}^{-1}$)$_{experimental}$	Yield of extraction ($mg_{carotenoids}.g_{dried\ mass}^{-1}$)$_{theoretical}$	Residues
1	0.017	65	0.02	1.56	1.33	0.23
2	0.035	65	0.02	1.57	1.74	-0.17
3	0.035	115	0.02	2.66	2.66	0.00
4	0.017	65	0.06	1.14	1.36	-0.22
5	0.035	65	0.06	1.23	1.45	-0.22
6	0.017	115	0.06	1.10	1.57	-0.47
7	0.017	115	0.06	1.98	1.57	0.42
8	0.035	115	0.06	1.14	1.95	-0.81
9	0.035	115	0.06	2.30	1.95	0.35
10	0.01088	90	0.04	1.09	1.18	-0.09
11	0.04112	90	0.04	1.21	1.84	-0.63
12	0.04112	90	0.04	2.40	1.84	0.56
13	0.04112	90	0.04	2.31	1.84	0.47
14	0.026	48	0.04	1.35	1.23	0.12
15	0.026	132	0.04	2.18	2.18	0.00
16	0.026	132	0.04	2.30	2.18	0.11
17	0.026	132	0.04	2.27	2.18	0.09
18	0.026	90	0.0064	2.33	2.37	-0.04
19	0.026	90	0.0064	2.27	2.37	-0.10
20	0.026	90	0.0736	2.01	1.80	0.21

21	0.026	90	0.0736	2.04	1.80	0.24
22	0.026	90	0.04	2.15	2.03	0.12
23	0.026	90	0.04	1.77	2.03	-0.26
24	0.026	90	0.04	2.04	2.03	0.01
25	0.026	90	0.04	2.04	2.03	0.01
26	0.026	90	0.04	2.03	2.03	0.00
27	0.026	90	0.04	2.02	2.03	-0.01
28	0.026	90	0.04	2.10	2.03	0.07

Table S6. Regression coefficients for the 2nd 2^3 factorial planning with surfactant Tween 20 aqueous solution.

	**Regression Coefficients**	**Standard deviation**	**t-student (10)**	**p - value**
interception	-2.63	2.276	-1.15458	0.263360
C_{surf}	129.37	78.836	1.64096	0.118163
t	-2276.44	1040.538	-2.18775	0.042123
$R_{(S/L)}$	0.04	0.033	1.33801	0.197553
C_{surf}^2	0.00	0.000	-1.38595	0.182695
t^2	18.28	32.247	0.56689	0.577793
$R_{(S/L)}^2$	48.57	197.483	0.24592	0.808525
C_{surf} * t	0.32	0.617	0.52025	0.609230
C_{surf} * $R_{(S/L)}$	-445.68	807.947	-0.55163	0.587988
t * $R_{(S/L)}$	-0.21	0.276	-0.76879	0.451986

Table S7. ANOVA table for the 2nd factorial planning 2^3 with surfactant Tween 20 aqueous solution.

	**Sum Squares**	**Degrees of Freedom**	**Mean Square**	**Fcal**	**p -value**
Regression	3.45	9	0.38	2.74	0.03
Error	2.52	18	0.14		
Total	5.97				

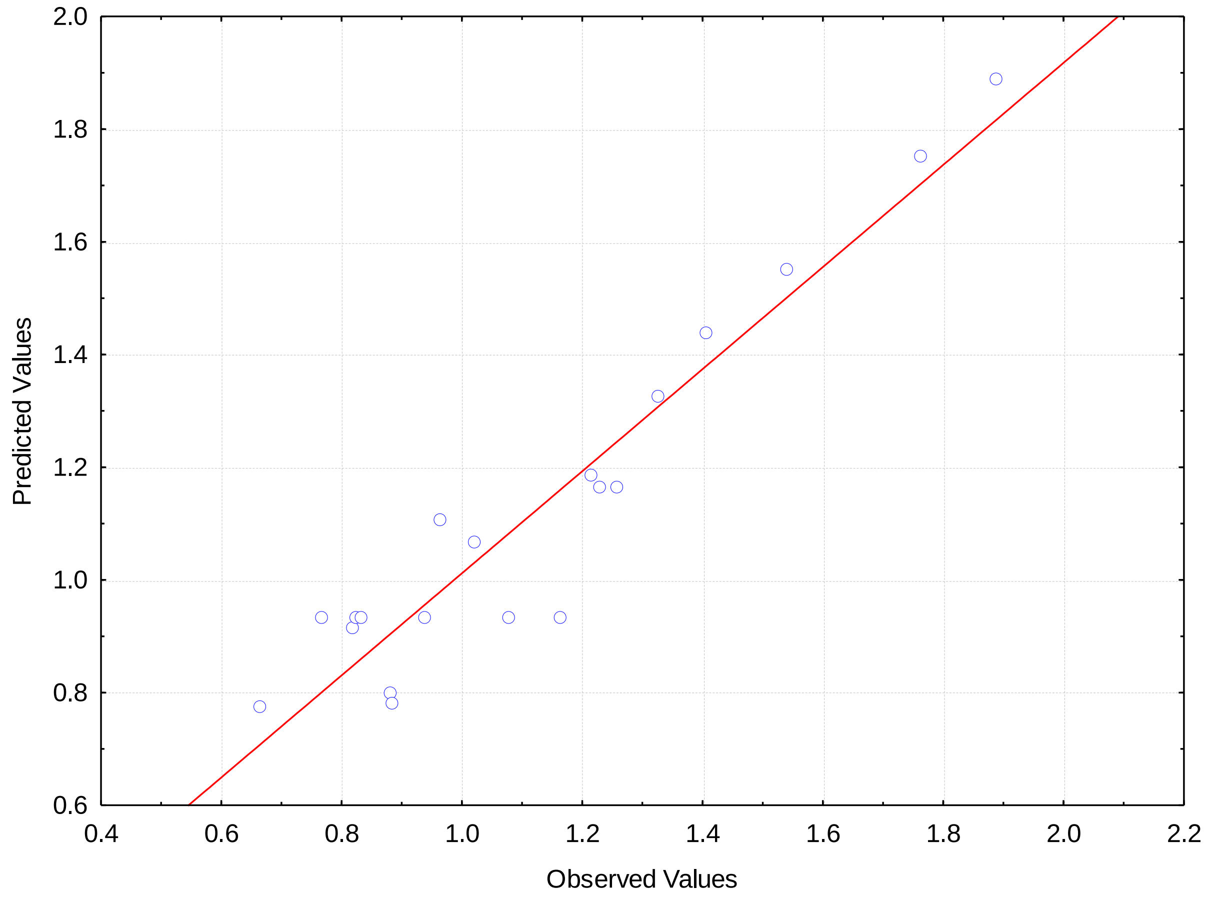

Figure S1: Graph for predicted values *versus* observed values for Tween 20, 1st RSM.

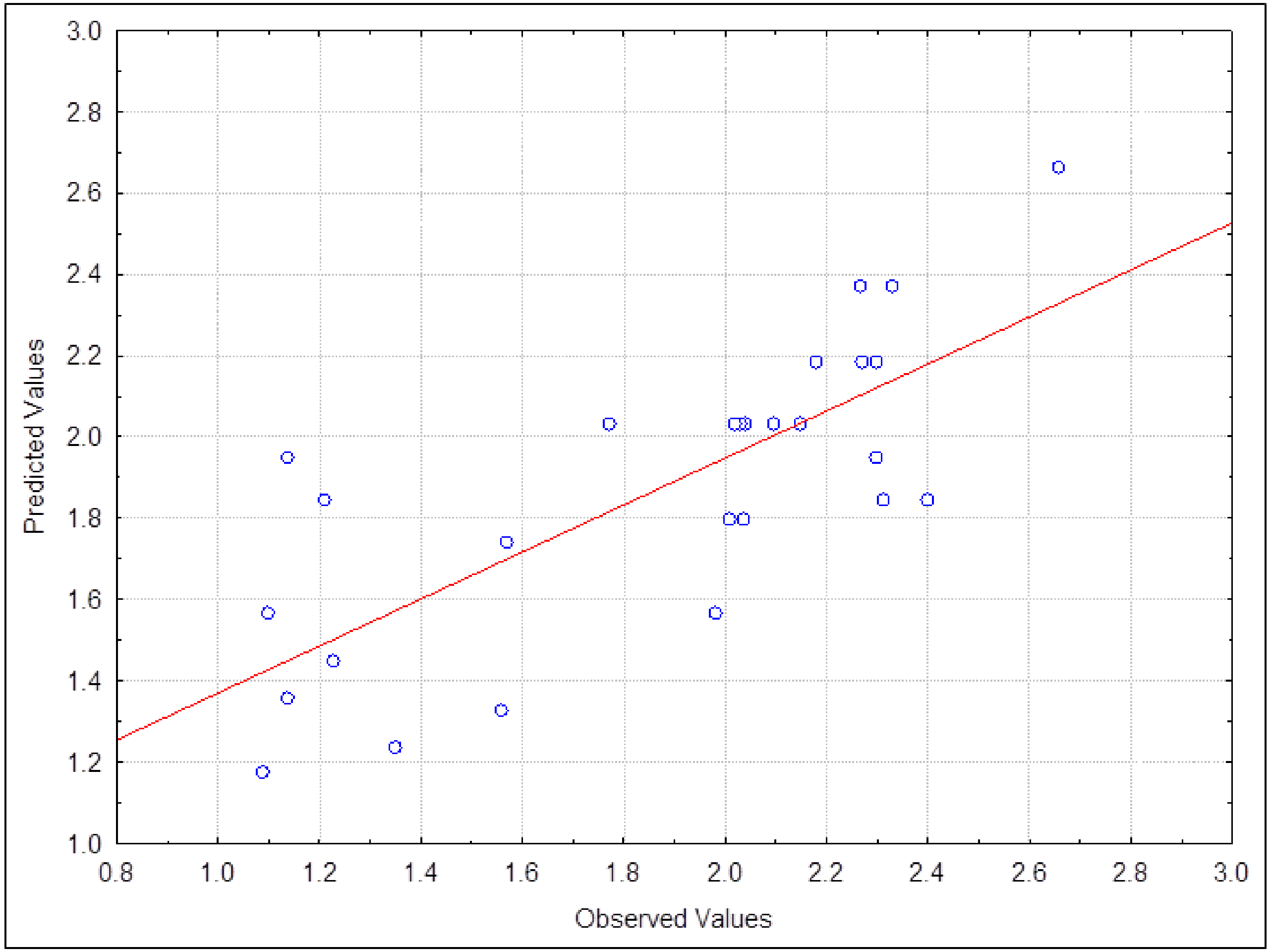

Figure S2. Graph for predicted values versus observed values for Tween 20, 2nd RSM.
